# Technological robot—Machine tool collaboration for agile production

**DOI:** 10.3389/frobt.2022.1027173

**Published:** 2022-10-25

**Authors:** Markus Wabner, Hendrik Rentzsch, Steffen Ihlenfeldt, Andreas Otto

**Affiliations:** Fraunhofer Institute for Machine Tools and Forming Technology, Chemnitz, Germany

**Keywords:** robot machining, collaboration, agile manufacturing, machining, matrix production

## Abstract

The flexibility and efficiency in parts production can be significantly increased through the technological cooperation of industrial robots and machine tools. The paper presents an approach in which a robot, in addition to the classic handling tasks, enhance machine tools by additional manufacturing technologies and thus beneficially supports workpiece machining. This can take place in various configurations, starting with pre- and final machining by the robot outside the machine, through sequential cooperative machining of the workpiece clamped in the machine, to parallel, synchronized machining of a workpiece in the machine. The approach results in a novel type of collaborative manufacturing equipment for matrix production that will improve the versatility, efficiency and profitability in production.

## 1 Introduction

The increasing customization of customer requirements, ever shorter product development and product life cycles, but also global crises confront manufacturing companies with the challenge of making their production more agile and more resilient to disruptive changes. Above all, agility and resilience in production means a high degree of flexibility and adaptability at all levels. The vision of agile manufacturing is not entirely new: “Agile manufacturing systems can be conceptually thought of as being an integrated whole of complex interacting sub-systems, organized in such a way as to endeavor towards a common set of goals” (Merchant, 1984). Even today it is a concept of economic success in production (Gunasekaran et al., 2019). Forced and enabled by the rapid progress in ICT and artificial intelligence, numerous novel solutions become possible to further enhance agility in manufacturing and production. Even if agile production is primarily a planning and control task, the functionality of the available manufacturing equipment is of crucial importance, since it must provide the necessary technological capabilities (skill level) in a highly flexible manner. Industrial robots are significantly contribute to automation in production and the enormous number of industrial robots result in low prices. This was a reason to increasingly use industrial robots for machining. Of course the functionality of standard industrial robots is limited in comparison to machine tools, especially regarding positioning and path accuracy, static and dynamic stiffness und thus process stability and dynamic path accuracy. With various ICT support and the use of NC control systems, industrial robots become more and more suitable for machining and increasingly substituting selected machining tasks.

Manufacturing cells with robot automation in particular offer extensive but still largely unused potential for improving agility and resilience in production up to novel equipment strategies for matrix production. This unused potential is seen in the flexible enhancement of machine tools by additional manufacturing technologies that are provided by combined handling and machining robots. While industrial robots are used today to automate machine tools (parts handling: loading and unloading) and remain in a waiting position during machining operations, in future the robots will also take on technological tasks instead of waiting. The aim is not only to use the unproductive phases of the robots in the future and thus increase the productivity of the entire system. It is much more promising and therefore more important to flexibly expand machine tools and machining cells with additional manufacturing technologies and thus significantly increase the overall flexibility and adaptability in matrix production.

## 2 Robot machining and collaboration

Industrial robots for robot machining are available from various companies. The series of HSM robots (HSM = High Speed Machining) of Stäubli is suited for precise high-speed metal machining such as deburring, polishing, drilling, thread cutting, prototyping or the reworking of weld seams of various materials (aluminum, stainless steel, composite, *etc.*). Accuracy is reached by absolute calibration, model-based error compensation, a self-developed NC control CS9 and special design of core components. Mabi Robotic is using direct encoders in the joints, which increases controllability and accuracy significantly and thus allows rough machining and finishing in milling, turning and other cutting technologies. The Mabi robots are using CNC SINUMERIK 840D sl from Siemens, and Siemens CNC control is applied increasingly to other robots for machining, e.g., Kuka, Comau or even Stäubli. The last mentioned systems are configured and delivered by various companies (e.g., Robot Machining, ibs automation, ARRTSM, Boll Automation, FerRobotics or Fill) to e.g., OEMs or die making industry. Special CAM solutions for robot machining are available (e.g., Tebis, robotized, moduleworks). Various research activities are dealing with the error compensation of industrial robots, e.g., with additional piezo actuators (Schneider, 2013) or model-based *via* control system (Sörnmo et al., 2012; Fu et al., 2020). A high precise industrial robot was developed in the Flexmatic project (Flexmatic, 2016), combining a number of approaches (very stiff design, sensor integration, various error compensations). A good overview about robot machining is also given by Ji (Ji et al., 2019).

Robot-machine cooperation is well-known from automation (handling robots). Technological collaboration, where industrial robots and machines simultaneously machine one part, are actually not known. Mitsubishi Electrics presented a NC control system which allows such a kind of robot-machine-collaboration, but also here a practical application is not known. Wieland Anlagentechnik GmbH (Wieland, 2017) has presented a simplified robot-machine-collaboration solution, where the fixture of a handling robot couples a part physically to guiding systems of a machine tool to increase accuracy and stiffness. Collaborative systems are known from robot-robot or machine-machine collaboration. For machining of weak rigid large thin-walled aerospace parts, so-called mirror milling systems are replacing traditional processing methods. A dual-robot mirror milling system consisting of a machining hybrid robot and a supporting hybrid robot is presented in (Xiao et al., 2019). The cutter and the flexible supporting head are installed at the end of the machining robot and the supporting robot. The wall thickness error is measured by ultrasonic and compensated by the machining robot for accurately controlling the machining thickness. A similar system for machine tools is described in (Zhang et al., 2019). Dual robot setup that is widely used in assembly and handling applications, is used by (Owen et al., 2004) for machining. Owen proposed a dual robot setup, with one robot handling the material and the second one bearing the tool. Due to the redundant degree-of-freedom, the authors designed an off-line programming system with an integrated algorithm to optimize the trajectories of the tool, using the pseudo-inverse method. The approach monitors torque in the robot axes while also finds the optimum configuration/poses to improve the accuracy of the final part by decreasing tool deflection and optimum absorption of machining forces. In (Lin et al., 2009) such a system is described for surface polishing.

In (Huang and Lin, 2003) a dual independent robot machining cell is described, where the programming development was carried out by using CAM software to generate cutter location data for 5-axis milling together with a post processor to translate the CL data to linear and rotational motions for the robot cell controller. The implementation of the dual robot setup was achieved by dividing the original CL data in two parts taking into account collision detection between the two robots and minimization of force generated inaccuracy of the final geometry. The author also developed an offline programming module, enabling off-line programming and simulation of the dual robot machining cell.

Optimal division and allocation of the work and performing path planning in a coordinated manner while considering the requirements and constraints of collaborative industrial robots system is addressed in (Hassan et al., 2019) for fiber placement tasks. A two-stage approach is proposed in this paper. The first stage considers multiple objectives to optimally allocate each industrial robots with surface areas, while the second stage aims to generate coordinated paths for the industrial robots.

## 3 Principle of the technological robot—machine tool collaboration

**FIGURE 1:**
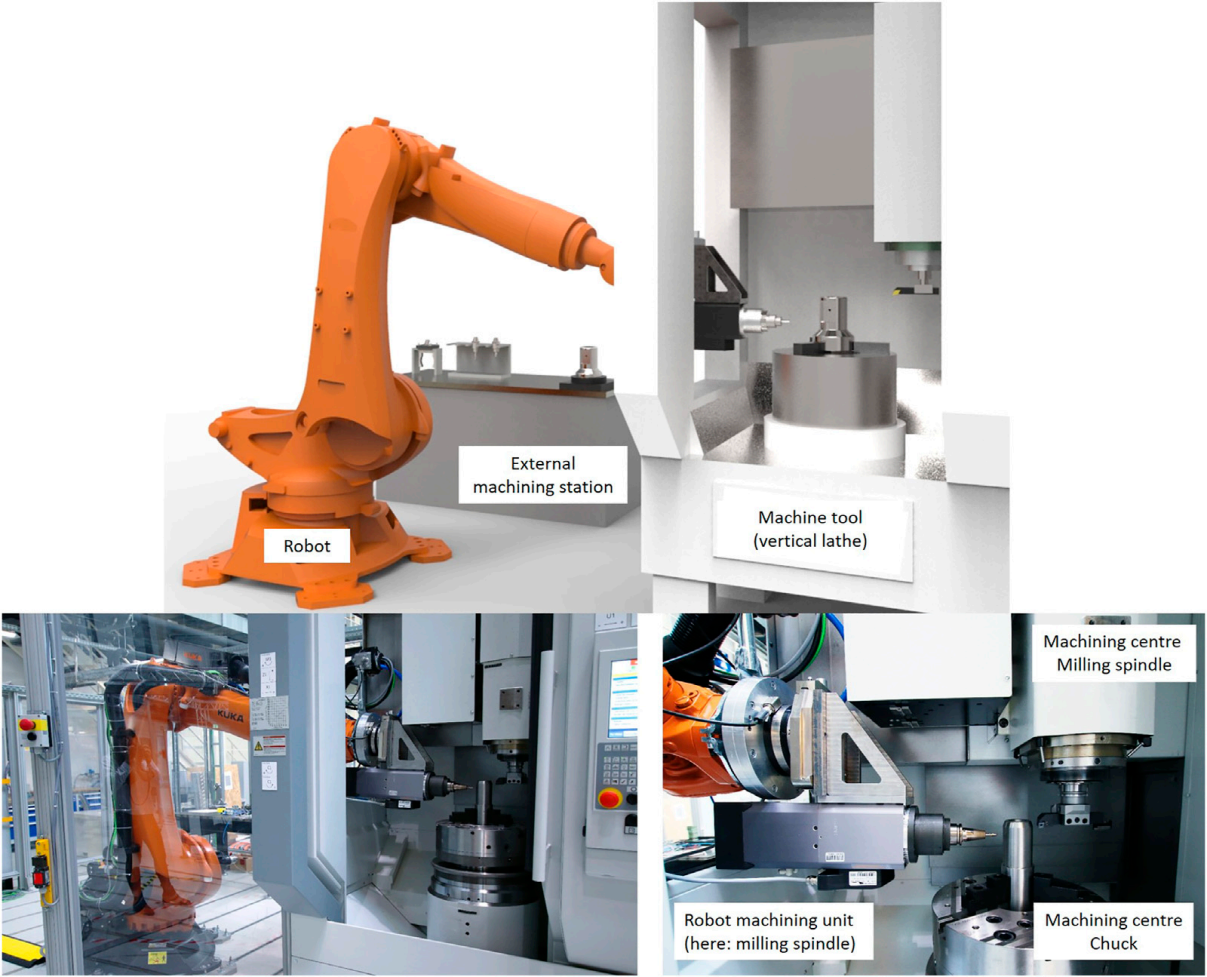
Principle of technological robot-machine-collaboration and authors test equipment.

Actually, the technological collaboration of machines and robots has been implemented only rudimentarily in industrial environments. But the technological enhancement of the limited functionality of machine tools offers a high potential for increasing productivity and a new equipment basis for matrix production making production more flexible with limited resources. In addition, under certain circumstances the range of functions of a machine tools and thus the investment can be reduced. As a side effect, the utilization of the robots also increases. In this way, machines can be expanded with missing or similar NC robot axes, for example to add missing rotary axes to a 3-axis machine or to machine a workpiece with two tools at the same time. Another example is the enhancement of machines with non-existent technologies (e.g., enhancement of milling machines with force-controlled grinding). The principle of the technological robot-machine tool collaboration is shown in Figure 1, using the example of the test equipment used by the authors.

The technological enhancement of machine tools can take place in three different scenarios:

- Sequential pre-processing or finishing of the workpiece outside the machine tool (e.g. robot-based removal of casting flash on the raw part; robot-based additive manufacturing; robot-based deburring or polishing on the finished part)
- Sequential machining of a workpiece within the machine tool (e.g. robot-based insertion of angled bores or execution of milling operations on a turned part clamped in a simple lathe)
- Parallel, synchronized machining of a workpiece by robot and machine tool (e.g. precision turning with machine, simultaneous robot-based deburring)

In addition to the technological scenarios mentioned as examples, the robots can of course continue to be used for conventional automation tasks such as handling or quality inspection. For this purpose, the robot must of course be equipped with the necessary technical systems (e.g. by changing systems).

## 4 Challenges and actual works

The realization of the robot-machine tool collaboration requires various research and development activities. It needs also novel strategies and fast CAM solutions to optimally break down a process chain to the involved systems. Such a CAM solution and the corresponding simulation tools are not available today. Furthermore, the strategy needs fast solutions for optimal (re-)configuration or (re-)allocation of the involved systems on the plant level as well as solutions for fast NC control coupling. To solve this, there is the need to describe the systems skills and to define skill levels of the systems. Together with technological and plant planning parameters, this will result in a very huge amount of thinkable combinations of machining and manufacturing scenarios. To handle the complexity and to find an optimal solution in an efficient way, AI-based optimization becomes a key technology. The main questions to be answered by optimization are.

- Which machining operation combination (or tool combination) would be the best?
- Which operation sequence would be the best?
- Which robot-machine combination should be selected?

**FIGURE 2:**
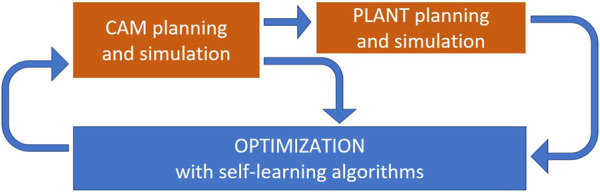
Optimization loops to find the best manufacturing scenarios.

And this under the consideration, that machine and robot are partly working together in parallel or in serial and that all machines and robots should preferable work at its maximum capacity. Thus, together with CAM simulation and PLANT simulation optimization circles (Figure 2) will be built to find the optimal parameter setup.

The inner optimization loop optimizes technological parameters, based on CAM simulation. The main optimization criterion is the machining time, which is also given to PLANT simulation. Here the various robot-machine combinations and its effects on the overall production of a plant will be simulated and optimised in the outer optimization loop. The used optimization algorithms are using artificial intelligence and are based on neural networks and genetic algorithms. The self-learning functionalities allow a significant reduction of needed optimization loops to find the optimum and its performance will increase by every new optimization task.

For the manufacturing optimization described, however, a number of other requirements must be fulfilled. One of them is the control system. It seems to make sense that each system retains its own NC control. However, the controller must be coupled together. This control coupling approach increases flexibility in production, both acutely and in the future by.

- Easy upgrade of existing machine tools and machining centres to a flexible production cell as basic equipment for matrix production
- Robots can be flexibly coupled to different machines, which increases the potential for versatility on the shop floor level
- The different systems can each be equipped with their optimal control

The control-related robot-machine coupling is implemented by coupling the two NC controllers using a Profinet (PN) connection (PN/PN coupling). The synchronization takes place both at the PLC level and at the NC level. Coupling at NC level is necessary to enable synchronized machining on a single workpiece. For this purpose, synchronization takes place at compile cycle level for synchronization in the position controller cycle.

An integrated CAM programming is the basis for the optimal splitting of the manufacturing tasks to the systems involved, for collision considerations and finally the generation of the G-codes. For this purpose, a CAM plugin for autodesk^®^ products is being developed in order to take into account two independent but simultaneously working tool systems. The assignment of necessary machining operations is feature-based. The feature approach is necessary as an orientation for the optimizer, which should nevertheless be open to external (digitized) planning suggestions in order to also incorporate the experience of operators and planners. The optimizer’s suggestion regarding the sequence, combination and parameterization of the machining tasks is then simulated in the CAM system. The result of the simulation is the digital output of key performance parameters (KPIs) for further optimization. The focus of the KPIs is on the processing time, which must be minimized. However, other criteria are also conceivable, such as energy requirements, tool wear or quality.

An essential prerequisite for the optimal distribution of tasks to the systems is a meaningful description of the technological capabilities (skill level) of the robot and machine tool systems. Ultimately, the question must be answered as to which processing task can be successfully carried out by which system? On the one hand, this requires the acquisition of system’s basic parameters such as workspace, spindle parameters, general performance parameters (forces, moments, feed speeds) or accuracies, which are usually specified by the system provider. On the other hand, special technological parameters such as accuracies that can actually be achieved, knowledge from experience or dependencies (e.g. accuracy-material removal rate) must be quantified. This may require experimental analysis or special processing tests. The recording takes place *via* standardized skill level protocols (e.g. “Administration shell” VDE/DKE), which must be digitally processable. This is a basic protocol that can be manually expanded and can ultimately represent part of a digital twin of a digital process chain.

## 5 Summary and outlook

In the article, a new strategy for the technological cooperation of machine tools and robots was presented in order to increase flexibility and agility as well as productivity in matrix production. However, the realization of such systems represents a major challenge and requires a large number of new solutions and planning methods, which the authors are currently working on. This includes AI-based algorithms for the optimal distribution of tasks between the systems both at workpiece and shop floor level, the system’s ability description (skill level), CAM-based tools for evaluating production scenarios and control solutions. The aim of the work is to implement a new type of flexible manufacturing cell that can be used both in classic parts manufacturing and as equipment for matrix production. If there are even several industrial robots and machine tools available, further optimization potential can be leveraged on the shop floor by the need-based and temporary combination of robots with corresponding machines.

## Data Availability

The original contributions presented in the study are included in the article/supplementary material, further inquiries can be directed to the corresponding author.
